# Comparison of light scattering-based detection methods for the sizing and number density characterization of extracellular vesicles (EV) isolated from human embryonic kidney (HEK) cell cultures

**DOI:** 10.1007/s00216-025-06310-3

**Published:** 2026-01-14

**Authors:** William F. Pons, Katelyn M. Joye, Terri F. Bruce, R. Kenneth Marcus

**Affiliations:** 1 Department of Chemistry, Biosystems Research Complex, Clemson University, Clemson, SC 29634-0973 USA; 2 Clemson Light Imaging Facility, Clemson University, Clemson, SC 29634-0973 USA

**Keywords:** EV isolation, Light scattering, Multi-angle light scattering, Nano-flow cytometry, Nanoparticle tracking analysis

## Abstract

Extracellular vesicles (EVs) are membrane-bound nanosized particles excreted by all cells and are of significant interest for biomedical applications such as diagnostic testing and as vectors for therapeutic delivery. EVs are abundant in biofluids, including urine, saliva, blood, and cell culture media, but must be isolated from their complex matrix for use. Once isolated, a primary challenge is determining the EV size distribution and the total number of particles recovered. Multiple detection methods are currently used to characterize EV recoveries in terms of sizing and number density determinations, the most common being nanoparticle tracking analysis (NTA), flow cytometry, and electron microscopy (transmission (TEM) or scanning (SEM)). Addressed here is a practical assessment of three common light scattering-based methods for the determination of EV population sizing and number densities. NTA, multi-angle light scattering (MALS), and nano-flow cytometry (nFCM) are directly compared. Specifically, the baseline practical advantages and disadvantages of each technique are evaluated via analysis of silica nanoparticle standards. Subsequently, EVs isolated from human embryonic kidney (HEK) cell culture supernatant using a hydrophobic interaction chromatography-based separation on the previously developed polyester (PET) capillary-channeled polymer (C-CP) fiber column platform were characterized. This isolation of high-purity EVs from HEK cell culture matrix components was validated using UV chromatograms, Bradford protein assays, SEM, TEM, and fluorescence nFCM analysis. The relative attributes of these important light scattering methods are presented in terms of their fitness for specific applications and overall effectiveness in EV size and concentration analysis.

**Supplementary Information:**

The online version contains supplementary material available at 10.1007/s00216-025-06310-3.

## Introduction

Extracellular vesicles (EVs), a class of biological nanoparticles, have shown great biomedical significance both as biomarker-carrying vehicles and for their putative use as drug delivery vectors for targeted therapeutics delivery [1]. Exosomes (30–150 nm), a subset of EVs, have a significant role in cell-to-cell communication and transport a variety of biological cargo, including proteins, lipids, RNA, and DNA, representative of their cell of origin [2]. Due to their low immunogenicity, EV-based therapeutics have high biocompatibility. In addition, the engineering of EVs enables augmented properties such as drug-loading efficiency and targeting efficiency for specific tissues [3]. EVs are released from all cell types and are present in all biofluids, including urine, milk, saliva, serum, plants, and cell culture [4]. Choosing an EV source depends on the desired application and is a balance between matrix complexity, EV concentration, and ease of biofluid/source collection, all of which are of consequence in terms of cost and scalability. EVs isolated from human embryonic kidney (HEK) cells have shown minimal immunogenicity and toxicity in mouse studies, making them a valid candidate for use in EV-based therapeutics [5, 6]. As such, HEK293 culture supernatant (a highly complex matrix) has been chosen as the EV source for this study due to the scalability and reproducibility of cell culture.

A recent edited volume presents a very broad overview of the analytical challenges in the study and utilization of extracellular vesicles and the relevant analytical methodologies employed in the field [7]. To that end, reproducible purification of EVs from their complex source matrices at scale is essential for downstream applications. EV purity is typically defined in terms of the number of EVs per µg of remnant protein, with a focus on isolating EVs from their matrix proteins [8]. EV isolations pose challenges due to the complex nature of their source matrix and similarities between the target vesicles and matrix components, specifically having a similar size and density, such as protein aggregates and low-density lipoproteins (LDLs) [9]. Currently, there are several methods used for EV isolation, including ultracentrifugation (UC)–based methods [10, 11], polymer precipitation [12, 13], size exclusion chromatography (SEC) [10, 14], and filtration methods such as tangential flow filtration (TFF) [15, 16] and asymmetrical-flow field-flow fractionation (AF4) [17]. While these methods are capable of isolating EVs for use in biochemical and diagnostic applications, each is typically hindered by high costs, lengthy separation times, and low yields when one considers scale-up to vector production levels.

Following EV isolation, accurate and reproducible characterization and vesicle verification are critical for downstream analysis and use. This is accomplished using various techniques targeting broadly: physical properties and biological activity. The typical physical properties of vesicle sizing and number density are commonly determined by transmission and scanning electron microscopy (TEM and SEM), nanoparticle tracking analysis (NTA), multi-angle light scattering (MALS), flow cytometry, and dynamic light scattering (DLS). EV detection and the determination of biological activity are investigated via immunolabeling techniques such as western blotting, fluorescent flow cytometry, and fluorescent microscopy. These techniques specifically target EV tetraspanins (commonly CD81, CD9, and CD63) and other biomarker proteins.

Light scattering (LS), in various forms, is a powerful approach for characterizing a wide variety of micro- and nanoparticles [18, 19]. The use of LS techniques in EV detection offers the potential for simultaneous size and concentration measurements of EVs in a suspension. Light scattering methods offer the potential advantages of minimal sample preparation and low volume/concentration requirements for analysis, conserving sample volume. Multiple factors contribute to the light scattering by particles, including particle diameter and refractive index, solvent refractive index, laser wavelength, and temperature, which must be considered and controlled when using these methods for particle suspension analysis [20]. Numerous LS approaches and platforms are currently used for EV analysis, with the most common methods being flow cytometry and NTA. However, less common instrumentation, such as multi-angle light scattering (MALS) and dynamic light scattering (DLS), has its advantages. The throughput of each method is important to consider, specifically if the detection of EVs is in line with the separation/purification process or performed afterward on EV isolate fractions. In this regard, MALS and DLS approaches offer high-throughput in-line detection, whereas NTA and flow cytometry require subsequent off-line analysis, post-collection.

Typical flow cytometers are designed for cell analysis and thus can detect larger EVs, such as microvesicles and large exosomes. Fluorescently labeling EVs lowers the limit of detection towards the detection of smaller EVs, although a major issue still present is that multiple smaller particles are likely to be detected as a single larger particle [20]. This can lead to overestimations of particle sizing and underestimations of particle number density. Modern nanoflow cytometers (nFCM) such as the CytoFLEX (Beckman Coulter, Brea, CA) and the flow nanoanalyzer (NanoFCM, Nottingham, UK) address these issues with the use of very low flow rates (10 s of nL min^−1^) and small sample volumes (~ 40 µL), allowing for single EV analysis with lower size bounds of ~ 40 nm, well within the needs for the majority of EV size determinations [21, 22]. For EV analysis, vesicles can be labeled with a variety of markers, both specific, such as CD9/CD81 tetraspanin protein markers, or non-specific, such as lipophilic membrane anchors, greatly increasing information content [23].

With NTA analysis, particles in suspension flow (~ 100 µL min^−1^) through a cell, where a laser induces light scattering from particles, and a camera equipped with microscope-scale lenses samples the intensity and frequency of light scattering [24]. This technique applies principles of the Stokes–Einstein equation to determine vesicle sizing, where using a solution of known viscosity, the rate of Brownian motion of the particle is inversely proportional to particle size [25]. Sample particle concentrations can be estimated based on sample flow rate and particles detected per image (particles per frame); however, a validation with a standard of known concentration ensures proper software estimations. Both nFCM [26–28] and NTA [29, 30] have been used extensively for EV detection and analysis for both size/number determination and for fluorescence analysis.

The MALS technique uses a single laser and multiple (e.g., 18) detectors arranged at different scattering angles to measure both the intensity and angular dependence of LS, enabling simultaneous sizing and number density analysis. This instrument leverages the Rayleigh–Gans–Debye theory of light scattering, which, in short, calculates particle sizing based on both the total intensity of LS and the relative LS at various angles [31]. The primary application of MALS lies in the characterization of polymeric materials, where effluents from size exclusion chromatography (SEC) are passed directly through the analyzer. The MALS technique for EV analysis is typically paired with SEC [32, 33] or tangential flow filtration [32, 34]. A previous comparison study using similar nFCM, NTA, and MALS instruments highlighted the use of these instruments in conjunction with SEC and AF4 for EV isolation from blood plasma samples [35]. MALS batch-mode enables the analysis of EVs from an unfractionated sample, providing both a particle size average and concentration determination.

This group has previously developed an EV separation platform that uses capillary-channeled polymer (C-CP) fibers as a stationary phase for hydrophobic interaction chromatography (HIC) separations in an HPLC column [36–38] and centrifuge spin-down tip [39–41] formats. Importantly, these fibers can be melt extruded into multiple geometries, most commonly a trilobal or an eight-lobed geometry. When C-CP fibers are pulled through tubing (typically polyetheretherketone (PEEK)), they efficiently interdigitate, creating a multitude of capillary channels spanning 1–5 µm in diameter, extending the length of the column. The interdigitation of fibers allows for efficient mass transfer and great surface area while maintaining relatively low backpressures and high linear velocities [42, 43]. The HIC method for the separation of EVs leverages the greater relative hydrophobicity of EVs in comparison to other matrix components [39]. A recent publication presents a comprehensive review of previous EV separations using C-CP fiber columns with the HIC modality, including various figures of merit, methods of validation, and benchmarking with other separation modalities [44].

Herein, a direct comparison of the NanoFCM flow nanoanalyzer (nFCM), the Malvern NS300 NTA, and the Wyatt (Waters) Dawn MALS highlights the specific practical advantages and disadvantages of each detection technique. The analysis of multiple silica sizing standards provides a fair comparison and benchmarking between each instrument’s capabilities and limitations. Subsequently, we employ the C-CP fiber platform in an HIC method for the isolation of EVs from an HEK cell culture supernatant, allowing instrument comparison for relevant sample analysis. Extensive supporting characterization validates the efficacy and purity of those collected fractions. This demonstration on an EV isolate provides for a pertinent evaluation of the performance of the instruments on a common sample, with the goal of informing the various use cases for each instrument for EV size/number density determinations. While one would hope that the methods of EV size characterization would be agnostic of the methods of the initial vesicle isolation, it must be admitted that there may be biases. That said, this two-step approach, characterization of standard materials followed by EV isolate characterization, better informs the use of the methods in the context of a singular EV isolate population.

## Methods and materials

### Chemicals and sample preparation

The HIC separation method solvents included ultra-pure ammonium sulfate (AS) and HPLC-grade acetonitrile (ACN), both purchased from VWR Chemical (Radnor, PA). Phosphate-buffered saline (PBS) at 10X, pH = 7.4, was purchased from Gibco (Grand Island, NY), and deionized water (DI H_2_O) was obtained from an Elga PURELAB flex water purification system (18.2 MΩ cm) (Veolia Water Technologies, High Wycombe, England). Four mobile phase compositions were used for the separations. First, a 2 M AS solution was prepared by dissolving AS in 1X PBS. Second, a 1 M AS + 10% ACN in 1X PBS solution was prepared by dissolving AS in 10% ACN in 1X PBS. Third, a 35% ACN in 1X PBS solution, where ACN was diluted directly with 1X PBS. Finally, the fourth solution was an 80% ACN in DI H_2_O column wash. The pH of all solvents was adjusted to 7.0 using HCl/NaOH (ACS grade, VWR Chemical, Radnor, PA) as needed. All mobile phases were filtered using 0.22 µm polyethersulphone (PES) 500 mL bottle top solvent filters (Thermo Fisher Scientific, Sunnyvale, CA, USA).

Human embryonic kidney cell (HEK) supernatant (from the exponential phase of growth) was provided by the Harcum Laboratory (Department of Bioengineering, Clemson University). The cell line used was the Gibco Viral Production Cells 2.0 HEK293F (Gibco, Waltham, MA), grown in Gibco viral production 2.0 media. Cytiva HyClone Cell Boost 5 Supplement (Cytiva, Marlborough, MA) was added to the media at 5% v/v on even days starting from day 4 (d4, d6, etc.); the cells were grown serum-free. Shake flasks (250 mL) were used and shaken at 175 rpm, in an incubator set at 37 °C and 5% CO_2_. Cells were removed from the growth media by low-speed centrifugation, 500 × g for 10 min. The sample used was a pooled sample of three biological replicates from day 14 of cell growth. On day 14, the viable cell density was ~ 16.7 × 10^6^ cells mL^−1^, with a viable cell percentage of 92%. These values were similar to a previous poster presented by the Harcum Group, demonstrating a viable cell density of ~ 16 × 10^6^ viable cells mL^−1^ on day 14 of growth [45]. All samples were subsequently prefiltered using 0.22 µm PES syringe filters (FroggaBio, NY, USA) and then frozen at −80 °C with a slow-freezing (−1.5 °C min^−1^) container until analysis. A lyophilized HEK EV standard was purchased from HansaBioMed Life Sciences (Tallinn, Estonia) and used as a concentration-based reference standard for comparison with the HEK supernatant isolated EVs. These EVs were reconstituted in 100 µL DI H_2_O and had an NTA calculated (from the manufacturer) concentration of 2.9 × 10^11^ particles mL^−1^.

### Chromatographic columns, HPLC instrumentation, and HIC method

All chromatographic separations used microbore columns with trilobal polyethylene terephthalate (PET-Y) C-CP fibers as the stationary phase. These fibers were provided by the Department of Materials Science at Clemson University. Fibers were packed into polyether ether ketone (PEEK) tubing (I.D. = 0.76 mm, “green PEEK”) as described previously [46]. In short, the PET-Y fibers are wound around a spool (*d* = 22 cm), where the number of rotations of fibers is selected to achieve an approximate interstitial fraction of ~ 0.6 as previously optimized [43, 46]. Specifically, six rotations were employed, resulting in 396 PET-Y fibers inserted through the column. Boiling DI-H_2_O was used to heat-shrink the PET-Y fibers, which were then pulled through the PEEK tubing and cut to a starting length of 32 cm. The column was washed with a standalone pump at 1 mL min^−1^ with DI water, 80% ACN, and DI water once more, each step for 5 min. After the washing steps, the fibers shrink slightly, and the ends are clipped to a final length of 30 cm. C-CP fiber columns were either immediately used or stored in 35% ACN in 1X PBS. PET-Y C-CP fiber column chromatographic performance characteristics, repeatability, and EV binding capacities have been characterized in a recent manuscript [47]. Of relevance to the EV isolations here, prior efforts have demonstrated the use of the HIC C-CP fiber methodologies specifically towards the isolation of HEK cell-derived EVs [45, 48].

A Dionex (Thermo Fisher Scientific, Sunnyvale, CA, USA) Ultimate 3000 HPLC system equipped with an LPG-3400SD quaternary pump and a VWD-3400RS variable wavelength UV–Vis absorbance detector (13 µL flow cell) was used for all chromatographic separations. The system was controlled using the Chromeleon 7.2 software system. For detection, absorbance at 216 nm was used to monitor the matrix protein and EV elution. Previous studies have demonstrated a linear relationship between the integrated absorbance (actually a measure of light scattering by the vesicles) and EV concentration at 216 nm, which has been applied in multiple instances to yield reproducible concentration determinations [39, 40, 49]. Absorbance detection at 254 nm and 280 nm was also used as a secondary means of monitoring protein elution. A mobile phase flow rate of 0.5 mL min^−1^ was used as previously optimized for the trilobal PET-Y fiber columns [37].

Before the HIC method began, the column was equilibrated in 2 M AS for 10 min (all flow rates are 0.5 mL min^−1^ unless indicated otherwise). The separation method began with a manual sample injection of 100 µL of 0.22 µm-filtered HEK supernatant using a Rheodyne 7725i injector (IDEX Health & Science, Northbrook, IL). The 2 M AS mobile phase was extended until time (*t*) = 3 min, where the mobile phase was changed to 1 M AS + 10% ACN, allowing mildly-hydrophobic species, such as proteins, to elute. At *t* = 6 min, the mobile phase was 35% ACN, allowing the more hydrophobic vesicles to elute. This was extended until *t* = 9 min, where a 3 min wash in 80% ACN at 1 mL min^−1^ continued until *t* = 12 min. Re-equilibration followed (after 1 min in 35% ACN to prevent AS precipitation) for 4 min at 0.5 mL min^−1^. All data was absorbance blank subtracted from a HIC workflow with no injection. Fractions of the peak of interest (protein and EV peaks) were manually collected in biocompatible microcentrifuge tubes when the UV signal increased at the expected elution point and held for 1 min (0.5 mL). After fraction collection, the ACN was evaporated by storing the open fraction vials at 5° C overnight, leaving the EVs in a PBS matrix.

### Light scattering instrumentation and comparison of methods

Three light scattering instruments were used for this study: the NanoFCM nanoflow analyzer, the Wyatt DAWN MALS instrument, and the Nanosight NS300 NTA instrument. For this comparison, all instruments were used to analyze two silica nanoparticle (SiNP) standards, as well as the fractions collected from the EV elution. (Note: In principle, the MALS determinations can be affected directly in-line with the LC eluate, but this approach was taken to provide a controlled environment, allowing direct comparisons between the base optical methods.) Critical to accurate EV LS measurements is the use of a standard with a similar refractive index to EVs. In this study, silica nanoparticles (RI = 1.43, nanoComposix, San Diego, CA) are used as the calibration standard for EVs, which have an estimated RI of 1.38 [50]. The first standard was NanoXact™, 80 nm SiNP (nanoComposix, San Diego, CA), acting as a reference/control with a single size population and known number density. The sizing/number density, as reported by the manufacturer, was 82 ± 3 nm and 1.6 × 10^13^ particles mL^−1^ (no measure of precision provided). This sample was diluted 1:10^4^ in DI H_2_O to fall within each instrument’s operating range. The second standard was a mixture of four distinct SiNP populations at 68, 91, 113, and 155 nm (nanoFCM, Nottingham, UK), used to analyze each instrument’s ability to distinguish multiple populations of particles simultaneously. Dilution was 1:100 in DI H_2_O, as recommended by the manufacturer. Finally, the EV elution fractions were analyzed on each instrument for real sample analysis, diluted 1:10 in 1X PBS in each case.

#### Nanoflow cytometer analysis

The nanoflow cytometer used in this study was the NanoFCM Flow Nanoanalyzer (NanoFCM Inc, Nottingham, UK), controlled by the NanoFCM Profession V2.0 software. In addition to the particle count and size distribution, which are determined by the scattering of the probe beam, the presence of CD81 tetraspanin membrane proteins was assessed with fluorescently-labeled anti-CD81 antibodies (Abs), and the presence of vesicular membranes was confirmed through a fluorescently-labeled lipophilic membrane anchor, thus providing complementary confirmatory information. (These capabilities are employed in this effort as confirmation of the efficacy of the HIC C-CP fiber isolates.) This instrument utilizes two lasers having outputs at 488 nm and 638 nm, with laser powers set at 6 and 20 mW, respectively. The two fluorescent emission filters have band passes of 525/40 nm and 670/30 nm, and the side scatter bandpass filter used was 488/10 nm. The equipped detector is a single-photon counting module (SPCM) with side scatter decay set to 10%, as recommended by the manufacturer. In addition to side scatter measurements, the EV samples were incubated with an anti-CD81 FITC Ab (monitored at 525/40 nm) (ExoBrite™ CD81 Flow Antibody, Biotium, San Francisco, CA, USA) and a red fluorescing membrane anchor (monitored at 670/30 nm) (MemGlow™ 640 nm, Cytoskeleton Inc., Denver, CO, USA). Both dye concentrations were optimized through a titration process, maximizing the signal-to-noise ratio, with a final dilution factor of 1:4000 for the anti-CD81 Ab and 1:2500 for the MemGlow. 

Calibration of this instrument is completed upon every startup using two SiNP standards provided by NanoFCM. The first is a particle density/fluorescent standard used for quality control of the instrument, aligning the lasers and lenses, and calibrating the particle density determinations. The second standard is the previously mentioned mixture of four sizing SiNPs, generating a particle sizing response curve. The particle detection threshold was set to “small threshold” on the instrument software, defined as S/N > 3, per the manufacturer’s instructions. The default sample acquisition time for this instrument is 60 s, which was used for the SiNP standards. The analysis time was reduced to 30 s for the HEK sample to reduce complex sample introduction time. Fluorescence measurement gating is accomplished differently by NanoFCM than by other flow cytometers. First, utilizing the low sizing limits of this instrument (~ 50 nm), a “side-scattering trigger” approach is used for fluorescent analysis. Particle side-scatter and fluorescence signal are recorded simultaneously, and only particles that have a significant side-scatter signal (> 3 × S/N) will be recorded. This allows signals from small fluorescent aggregates to be excluded from those of the particulates, thereby reducing the false positive signals [21]. Particles that show fluorescence intensity exceeding the fluorescence signal-to-noise ratio are displayed as a function of both height (peak intensity) and width (integration of intensity over time for a single event). Fluorescent negative (below S/N) particles are only defined by the area of background diluent fluorescence. This calculation allows for a visual separation of the positive and negative populations. This “gating strategy” is typical for NanoFCM measurements and has been used for EV measurements previously [26, 51].

#### Nanoparticle tracking analysis

The Malvern NanoSight NS300 (Malvern Panalytical, Great Malvern, UK) was used for all NTA measurements in this study. This instrument is equipped with a single 488 nm laser, controlled by NanoSight NTA 3.4 (build 3.4.4). Again, with this instrument, the two SiNP particle samples and the EV sample were measured and repeated three times for each sample. Blank injections of water and the EV isolation solvent (1X PBS) resulted in little background contribution to the measured scattering signal. The flow cell was washed (> 1 min) with DI-H_2_O and 70% EtOH between samples. Major operational parameters used for the instrument were: camera level = 12–14, frame rate = 25 fps, and an integration time of 60 s. The camera level was modified on a sample-to-sample basis to reduce background to an appropriate level, minimizing non-particle detection. The solution viscosity, as calculated by the instrument, was 0.925 cP, based on the flow cell temperature and water as the solvent. The detection threshold was minimized without allowing the background signal to contribute significantly, which corresponded with the software setting of “5.” Measurements were taken at ambient temperature, 23.3 °C. The standard-equipped sCMOS camera was used. The instrument method began with a 20 s sample purge at 1 mL min^−1^, followed by a 120 s sample load at 100 µL min^−1^. To generate suitable statistics, five sets of 60 s acquisitions followed at a flow rate of 100 µL min^−1^.

#### Multi-angle light scattering analysis

The Wyatt DAWN™ (Waters/Wyatt, Goleta, CA) MALS instrument was used for all MALS analysis and was controlled by the ASTRA 8 Software. Again, for the sake of a more direct comparison with the other methods, the MALS was operated as a standalone unit versus direct integration with the C-CP fiber column HPLC output. Two calibration procedures were completed before sample analysis, following the ASTRA 8 Software User’s Guide. The instrument detector normalization procedure was completed using bovine serum albumin (BSA) protein dissolved in water. The LS calibration procedure was completed using the recommended solvent, 100% toluene. Important instrument operational parameters were: flow rate, 0.5 mL min^−1^; solvent, 35% ACN (RI, 1.348); injected volume, 100 µL; calibration constant, 6.0538 × 10^–5^ (V cm)^−1^; sphere real RI, 1.38 for EVs; 1.43 for SiNP; and EV dn/dc, 0.185 mL g^−1^. The exact dn/dc (refractive index increment) for EVs is unknown, but Wyatt recommends using the virus-like particle dn/dc of 0.185 mL g^−1^ as an estimate. This is consistent with multiple sources estimating the dn/dc of EVs to be approximately ~ 0.18 mL g^−1^ through relative protein (dn/dc: 0.19 mL g^−1^), RNA (0.18 mL g^−1^), and phospholipid (0.16 mL g^−1^) content [52, 53]. Sample fractions and standards were injected using a Rheodyne injector. The Debye scattering model was selected for particle sizing analysis, as it can detect both smaller (~ 30 nm) and larger EVs (greater than ~ 100 nm). A fit model at a power of 2 provided the most consistent results. The sphere model was selected for the analysis of number density. The high-angle detectors (i.e., 1–3 and 16–18) were excluded as needed if the signal contained high noise was not reproducible or was out of range. Fitting models and detector exclusion followed Wyatt recommendations.

### Scanning and transmission electron microscopy sample preparation and imaging

Scanning electron microscopy (SEM) imaging was used to visualize a C-CP fiber column cross-section, a single C-CP fiber, and EVs bound to the C-CP fiber surface. The cross-section samples were prepared by freezing the column in liquid nitrogen for ~ 10 s and immediately cutting cross-sections with a fresh razor blade. The fiber side view and EV images were prepared by first loading HEK supernatant on the column (~ 1 mL of sample loaded) under the 1 M AS + 10% ACN protein elution conditions (i.e., EV retained on-fiber). After loading, a ~ 1 cm section of the column was cut, and the fibers were removed and carefully spread across a carbon-taped SEM plate. The samples were placed in a Hummer 6.2 Sputtering system (Anatech USA, Union City, CA), coating all samples with platinum. The samples were placed in a vacuum chamber, and the pressure was reduced to ~ 40 mTorr. The chamber was filled with 200 mTorr argon, then reduced to 80 mTorr. Voltage was increased until the current was 15 mA. The samples were coated for 1.5 min each. The samples were desiccated for 30 min and then imaged using a Hitachi Regulus 8230 (Hitachi, Tokyo, Japan) at the Clemson University Electron Microscopy Facility with the following conditions: voltage of 10 kV, beam current of 22.3 µA, lens mode high, and condenser lens #2.

Transmission electron microscopy was conducted at the University of Georgia, Georgia Electron Microscopy facility, with a JEOL 2100Plus microscope (JEOL, Tokyo, Japan) operating at an accelerating voltage of 120 kV. A 3% phosphotungstic acid (PTA) stain at 6.8 pH was used for EV negative staining. A 5 µL droplet of EV isolate was placed on the coated side of a fresh 200-mesh copper carbon/formvar coated grid (Ted Pella, Redding, CA), allowed to rest for 15 min, and then gently blotted away with filter paper perpendicular to the grid. A droplet of PTA stain (~ 50 µL) was placed onto a piece of parafilm, and the grid was flipped sample side down onto the stain for 15 s and blotted with filter paper. The samples were air-dried for 15 min and then were imaged.

### Bradford assay analysis

Bradford protein assays were used to quantify the background protein content throughout the HIC separation. Values were determined for the raw HEK supernatant as well as the protein and EV elution fractions of the HIC EV isolation process to monitor the efficacy of the HIC method for latent protein removal. The protein and EV fractions contain 10% and 35% ACN, respectively. To remove ACN, all samples were allowed to evaporate overnight at 5 °C. After ACN evaporation, the sample volumes were corrected to 1 mL with 1X PBS. As a protein calibration standard, a primary bovine serum albumin (BSA) standard (2 mg mL^−1^ in DI H_2_O) was used (Albumin Standard, Thermo Fisher Scientific). Calibration solutions were prepared by dilution in 1X PBS to generate a response curve spanning 1–100 µg protein mL^−1^. Pierce™ Bradford (Coomassie) Plus Protein Assay Reagent (Thermo Fisher Scientific, Waltham, MA) was used for the colorimetric reaction. After the addition of Bradford reagent to all samples, the 96-well plate was inserted into a BioTek SynergyLX multi-mode reader (Winooski, VT). The plate reader initiated a 30-s agitation step, followed by a 10-min incubation period. Triplicate (*n* = 3) absorbance measurements were made at 595 nm, with a 30-s mixing step between each measurement.

## Results and discussion

### Comparison of LS instrument performance towards particle sizing and density determinations of standard materials

When designing comparison methods for the three instruments used to characterize EV separation isolates, it was critical to evaluate each instrument’s ability to measure sizing and number density accurately at EV-relevant sizes and concentrations.

**Fig. 1:**
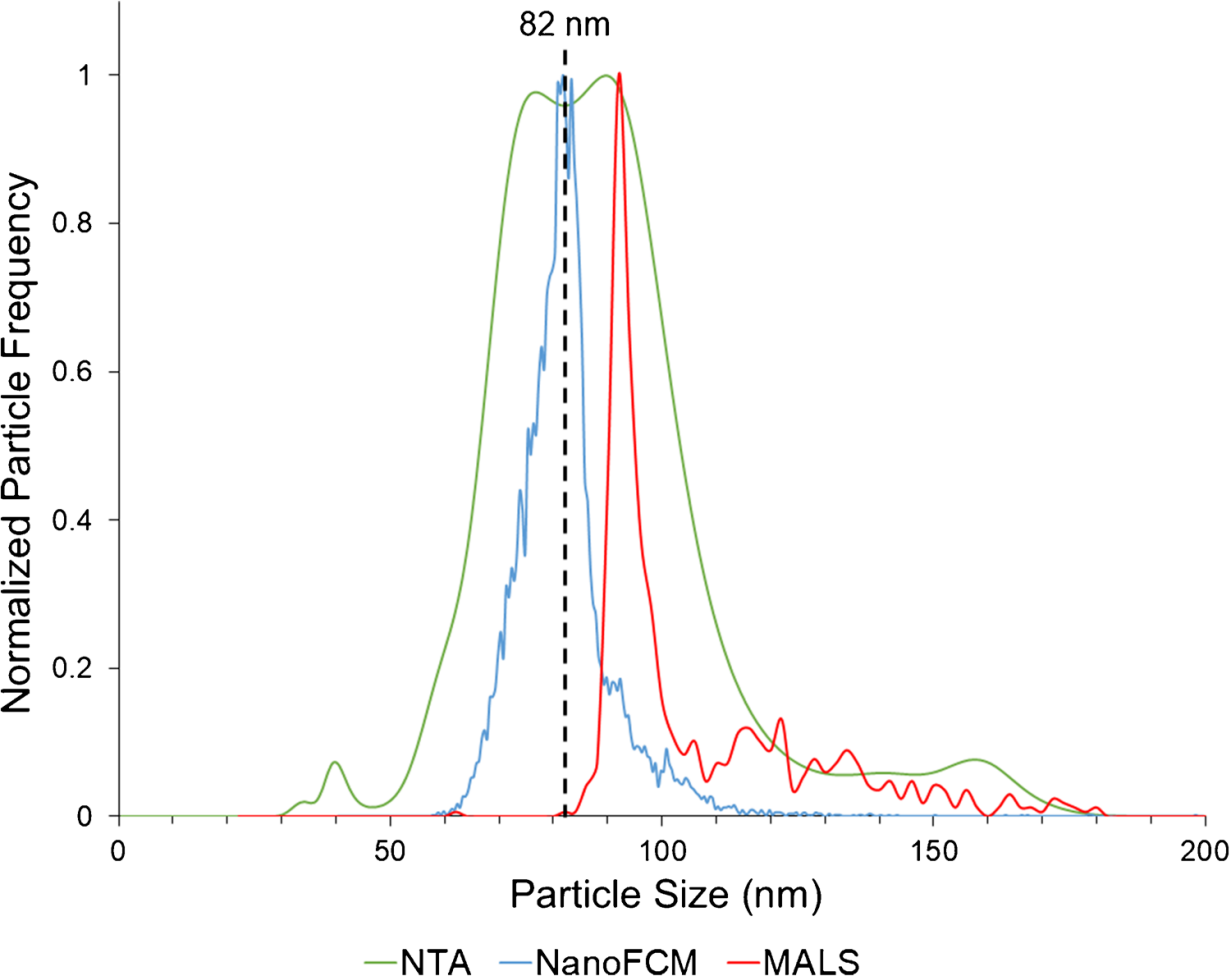
Overlay of average (*n* = 3) particle size distributions (PSDs) derived from the LS instruments using a single population of 82 nm SiNPs with a stated concentration of 1.6 × 10^9^ particles mL^−1^

**Fig. 2:**
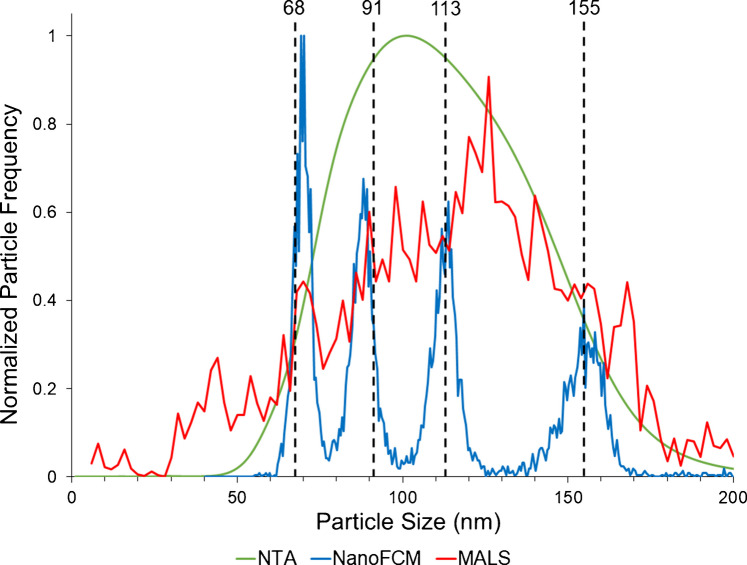
Overlay of average (*n* = 3) particle size distributions (PSDs) derived from the LS instruments using a mixture of four populations of SiNPs, including 68, 91, 113, and 155 nm. Note: MALS PSD has been shifted –15 nm based on the use of the single-population standard

It cannot be stated more strongly that any such comparison of methods suffers greatly from the fact that there are no certified or standard reference materials (CRM or SRMS) in the realm of EV analytics. As such, the industry-accepted silica nanoparticles (SiNPs) were used for the benchmarking. This overall comparison was completed in three parts: first, a standard of a single population of particles at a known size and concentration; second, a standard of four populations of particles at known sizes; and third, a real sample of EVs isolated from HEK supernatant. For each of the following analyses, robust controls were required to minimize potential signal contributions from the solvent. In all cases, a 1X PBS control (filtered with a 0.22-µm syringe filter) was used to ensure the background signal was minimal. Additional controls were used as stated in each corresponding section, depending on the technique and manufacturer recommendations. The size distribution data presented in Tables 1 and 2 and Figs. 1, 2, and 5 are averages of triplicate trials (*n* = 3).

#### Baseline performance evaluation using 82 nm SiNPs

The first instrument comparison was for a single-population standard of 82 nm SiNPs, representative of the general range of typical EV sizes. As previously discussed, SiNPs were chosen due to their similar refractive index to EVs. Figure 1 presents comparative particle size distributions (averaged from three determinations) obtained from each of the three instruments, with an indicator bar at the 82 nm manufacturer-provided mean for these particles. Qualitatively, the particle size distributions (PSDs) differ among the methods. First, the PSDs obtained from the nFCM and MALS instruments are very narrow, albeit only the former is centered about the projected particle diameter. On the other hand, while centered about the expected diameter value, the PSD generated by the NTA instrument is quite broad and seems to have some bi-modal character. Table 1 presents the quantitative measures derived for each method, as well as the manufacturer-provided values for the reference standard. It is noteworthy that the provided particle number density was derived from TEM data, with no indication of the measurement precision, and so it is difficult to assess the validity of the value.

**Table 1:** Quantitative results among the three light scattering test methods for *n* = 3 determinations of 82 nm silicon nanoparticles (SiNPs). Manufacturer particle size and number density (after dilution) were 82 ± 3 nm and 1.6 × 10^9^ particles mL^−1^ (no measure of precision provided)

Instrument	Avg. diameter (nm)	Median diameter (nm)	Avg. full width at half max (nm)	Avg. number density (particles mL^−1^)
MALS	97.7 ± 2.5%	96.7	6.0	2.12 × 10^8^ ± 2.1%
nFCM	80.5 ± 5.6%	81.3	10.5	5.32 × 10^8^ ± 0.3%
NTA	82.1 ± 15.1%	86.0	37.0	2.67 × 10^8^ ± 4.3%
TEM (manufacturer)	82 ± 1.8%	-	-	1.6 × 10^9^

As would be anticipated based on the PSDs of Fig. 1, the average (and median) particle diameter values determined by the nFCM and NTA instruments agree quite well with the provided value, although the nFCM provided appreciably better measurement precision and accuracy, which is reflected as an increased median in the NTA measurements. Alternatively, the median value derived from the MALS analysis, while precise, is ~ 15 nm (~ 20%) overestimated. Beyond the average and median diameter values provided by the three LS test methods, the full width at half max (FWHM) values provide a distribution broadening statistic. Within a “single” particle population, sizing performance should be based on minimizing this value, i.e., the smallest distribution about 82 nm. In this regard, the breadth of the MALS (6.0 nm) and nFCM (10.5 nm) PSDs were comparable, while NTA (37.0 nm) yielded a significantly broader distribution.

As noted in the analysis of the 82 nm SiNP sample, a ~ 15 nm positive bias was observed to exist in the MALS measurements. A similar bias was reported where MALS showed a larger size in comparison to SEM data for a similar SiNP material [54]. MALS (and NTA) measure particle hydrodynamic radius; particle hydration layer attributes will affect these techniques to a greater extent than a fixed angle detection method such as nFCM. This hydration shell interference was also postulated for the results of a polydisperse standard and EV isolates in a MALS analysis [34]. Another source of possible overestimation is the non-linear scaling of scattering intensity (intensity scales with r^6^). Specifically, when heterogeneous particle sizes are present and measured simultaneously, the average particle size may be overestimated based on the larger-sized particles. Based on the observed offset, for what is a well-defined standard, a –15 nm shift was employed in the plotting of subsequent MALS PSD data in this effort (Figs. 2 and 5). The efficacy of this correction is demonstrated by the agreement between the MALS distributions determined here for the second standard and the EV PSDs for the other two methods. The NTA instrument overestimated the SiNP by only a slight margin and was not adjusted; however, the increased broadening seen with this instrument may be due to multiple factors including uncertainty in particle position, limitations in particle tracking (short tracks/particles), and interactions with the flow cell walls (“rolling” particles). Any of these limitations may cause population broadening due to increased sizing uncertainty [50]. The nFCM analysis results were similar to the manufacturer-provided TEM measurements.

The second degree of performance important in this comparison was the ability of each instrument to accurately provide particle densities. The average number densities determined by each technique are compared to the reported TEM value from the manufacturer in Table 1. As seen here, the nFCM number density was ~ 3 × less than reported in the SiNP data sheet, with the values derived from the MALS and NTA determinations being ~ 6 × less than expected. Perhaps not surprisingly, the measurement precision for the particle number densities was appreciably better for all the techniques than for their corresponding sizing experiments, with the nFCM yielding a truly excellent 0.3% variability across triplicate (*n* = 3) measurements. That said, the universal trend might suggest that the particle density validity of the primary material assay may be in question; however, relative instrument-to-instrument particle density comparisons demonstrated particle densities well within an order of magnitude.

#### Baseline performance evaluation using a mixture of four SiNP size populations

The ability to better discern subpopulations of EVs is of growing importance as the roles of different size fractions, particularly those referred to as ‘small EVs’ (sEVs), are being realized [55, 56]. After determination of the single-population characteristics, the performance of the LS instruments was compared using a SiNP cocktail. The commercial assessment mixture contained four populations of SiNPs (68, 91, 113, and 155 nm), spanning the typical size range for EV (the manufacturer provides no quantitative statistics regarding sizing or concentrations). The average PSDs (*n* = 3) derived from each of the three instruments are presented in Fig. 2 (incorporating the –15 nm shift for the MALS data). The differences among the three instrumental methods are indeed quite pronounced, with only the output of the nFCM representing four distinct populations, whose centers agree very well with the expected sizes as indicated by the dashed vertical lines. In fact, each of the subpopulations in the mixture is clearly defined, and the baselines are resolved. As might be expected, there is some degree of population broadening with increased particle size. In the case of the MALS determinations, there is some level of structure in the PSD, though exact assignments would be very difficult to make. With MALS batch analysis, multiple particles are measured simultaneously and their sizes averaged; a broader distribution is expected. In this case, a larger size bias and greater noise were seen in comparison to the other two methods. The PSD derived from NTA showed a singular, very broad population, which was somewhat surprising from a single-particle analysis technique. The Gaussian-like distribution shown in Fig. 2 is much like that of Fig. 1 for the single-population case. The lack of finite structure in both NTA distributions is very similar to a previous study, which compared nFCM to NTA responses to both silica and polystyrene nanoparticle cocktails [57]. Overall, the NTA instrument showed a diminished sizing resolution in comparison to the other single particle analysis instrumentation, nFCM.

### Validation of the efficacy of the HIC C-CP fiber isolation process

As is true in all forms of chemical analysis, the quality of the information obtained is only as good as the quality of the input materials. In this regard, the separation methodologies employed in the EV isolation must yield particle suspensions of the highest purity, while also ensuring that the vesicles have also retained their primary physical and biological attributes. For the purposes of this comparison study, it was imperative to prepare an EV isolate of significantly high purity for a just comparison of the LS detectors. Indeed, the presentation of an EV isolate of poor purity (i.e., the presence of aggregates and lipids) is a primary limitation in all means of sizing/counting. The ability to consistently deliver high-purity EVs is a particularly positive trait of the C-CP fiber isolation methods [41, 49, 58]. In this effort, the goal was not to prove the absolute biological efficacy of the specific EV isolates, but rather to generate a compositionally clean population for LS characterization. To this end, multiple analysis methods were used to ensure that the EV elution was of sufficient quality for further analysis, including UV absorbance, evaluation of protein removal characteristics, SEM/TEM imaging, nFCM immunoconfirmation, and finally, the LS comparison.

#### EV chromatographic isolation and matrix protein removal

**Fig. 3:**
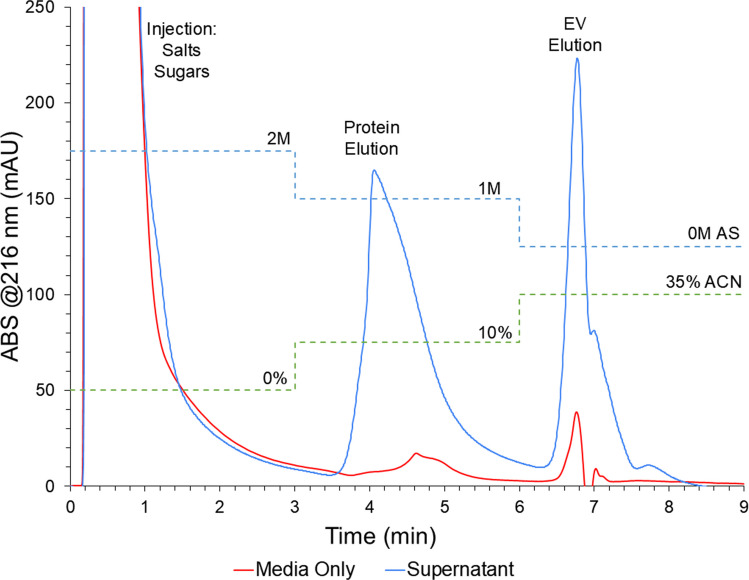
UV absorbance chromatograms demonstrating the HIC gradient program employed for the isolation of EVs from culture supernatants using a PET C-CP fiber column. Traces represent averaged chromatograms (*n* = 3) of 100 µL injections of fresh (unused) cell culture media (as a control) and cell culture supernatant

The chromatogram obtained from the HIC separation with a PET C-CP fiber column is shown in Fig. 3. The three separation steps are labeled as: injection, the 2 M AS injection condition; protein elution, the 1 M AS + 10% ACN protein elution condition; and the EV elution, 35% ACN. Fresh cell culture media (CCM) was used as a control for this separation. At the start, 100 µL of HEK cell supernatant or CCM control was injected into the 2 M AS mobile phase, where significant UV absorbance was seen in the injection/void volumes due to the elution of unretained components present in both matrices. The similar injection profiles reflect the abundant salts, amino acids, and sugars in the CCM, along with HEK metabolites in the culture, which are not retained on the column. The first elution step of 1 M AS + 10% ACN represents a reduction in ionic strength along with a contribution from the organic solvent, resulting in the elution of the more hydrophobic proteinaceous material. As expected, a significant contribution from protein products in the HEK supernatant is prevalent, and the media-only control had only a minor contribution from proteins added as a minor component in the CCM.

Finally, the EV elution step follows, where the mobile phase salt content is solely PBS, and the ACN concentration is increased to affect the EV release. A significant response is seen in the supernatant analysis, and a very minor elution response is seen in the media-only trials. The minor contribution seen from the CCM in the EV window may be partially due to lipids added in the serum-free CCM.

The removal of latent protein from the HEK supernatant sample matrix is crucial for isolating EVs for proteomics analyses and downstream applications. To ensure that the HIC isolation process successfully removed free protein contaminants, a Bradford protein assay was performed. Supplementary Information (Fig. S1) illustrates the successive reduction of the protein content, evolving from the raw supernatant to the protein elution fraction and the final EV eluate. The Bradford (Coomassie) reagent reacts primarily with basic amino acids (prevalent in the CCM feedstock) and so will bind with both latent (free) proteins and exposed proteins (e.g., tetraspanins) found on the EVs’ surfaces. The BSA response curve from 1 µg protein mL^−1^ resulted in an *R*^2^ of 0.9972 with a calculated LOD of 1.18 µg protein mL^−1^. The results of triplicate (*n* = 3) determinations of the raw sample yielded an average value of 596 ± 13 µg protein mL^−1^, > 110 times that of the EV eluate, with 36.4 ± 8.1 µg protein mL^−1^ found for the protein elution fraction and 5.4 ± 0.5 µg protein mL^−1^ in the target EV fraction. The trends observed here reflect the significant removal of protein prior to the final collection of EVs, with the variability in the EV fraction values (~ 5% RSD) indicating a very high level of process reproducibility. It is important to note that the final protein content seen from these isolations was comparable to previously reported protein assays for HIC/C-CP fiber EV isolations [59, 60].

#### SEM and TEM characterization of EVs

Scanning electron microscopy (SEM) was used to visualize the C-CP fiber column geometry as well as the adsorption of EVs onto the fibers themselves. A low-magnification view of the PET C-CP fiber column is seen in SI Fig. S2a, where a cross-section of the column was prepared. The interdigitation of the trilobal polymer fiber, which spans the length of the 30 cm column, is visible. In this experiment, the surface of the fibers was exposed to EVs through multiple injections of HEK supernatant (five injections of filtered 100 µL supernatant) into the protein elution mobile phase, affecting EV adsorption and protein elution simultaneously. SI Figs. S2b–d visualize this condition, shown in order of increasing magnification. In SI Fig. S2b, a single fiber (approximately 30 µm across) is removed from the EV-loaded column, where a groove is seen on the side of the fiber, and multiple micron-sized particles are seen bound to the fiber surface in this micrograph. These crystalline/jagged particles are likely salt crystals, as the solvent employed at this point in the process contains 1 M AS. Based on the sizing, the “soft/round” particles seen across the surface are likely due to aggregates of proteins or EVs occurring when the column is overloaded. That said, the potential for distortions, whether affected in the sputter-coating process or upon subjection to high vacuum, must be acknowledged. It is important to note that the presence of such micron-sized aggregates is not seen in the EV eluate fraction by any of the applied LS techniques, wherein less sample is loaded and the fiber is exposed to the EV elution solvent. In fact, none of the instruments show appreciable populations of > 200 nm particles in the final eluates, demonstrating that the dilution and storage conditions did not influence particle aggregation.

Once the magnification is increased to > 10,000 × in SI Fig. S2c, EVs are seen bound to the fibers in great numbers. The valley of the single fiber can be seen as a change in shadowing across the micrograph. As magnification is increased to 35,000× (SI Fig. S2d), the sizes of individual bound EVs become apparent. The vesicles span the expected 30–150 nm range for sEVs, with a minor population of particles expanding to a maximum of ~ 200–300 nm. The spherical-shaped vesicles bound to these fibers are consistent with previously reported SEM micrographs, highlighting the adsorption of EVs to the C-CP PET fibers [39, 61].

TEM is a powerful EV imaging technique commonly employed for verification of EV morphology, particularly analyzing an intact membrane, typically having a spherical or “cup-shape.” Presented in SI Fig. S3 are two TEM micrographs of EVs isolated from HEK supernatant with a HIC separation. Between the two micrographs, vesicle sizes ranging from approximately 40 to 150 nm in diameter are very evident. Multiple vesicles are seen with diverse topologies, with intact vesicle membranes seen in all highlighted vesicles. The EV labeled at the bottom-right of SI Fig. S3b features a typical cup shape. Non-EV contamination appears to be minimal, with much of the background being contributed to the PTA staining itself (dark spots, uniform staining “dots” seen throughout). Overall, numerous EVs can be visualized in these micrographs, spanning the typical size range of small EVs.

#### nFCM fluorescent immunoconfirmation

**Fig. 4:**
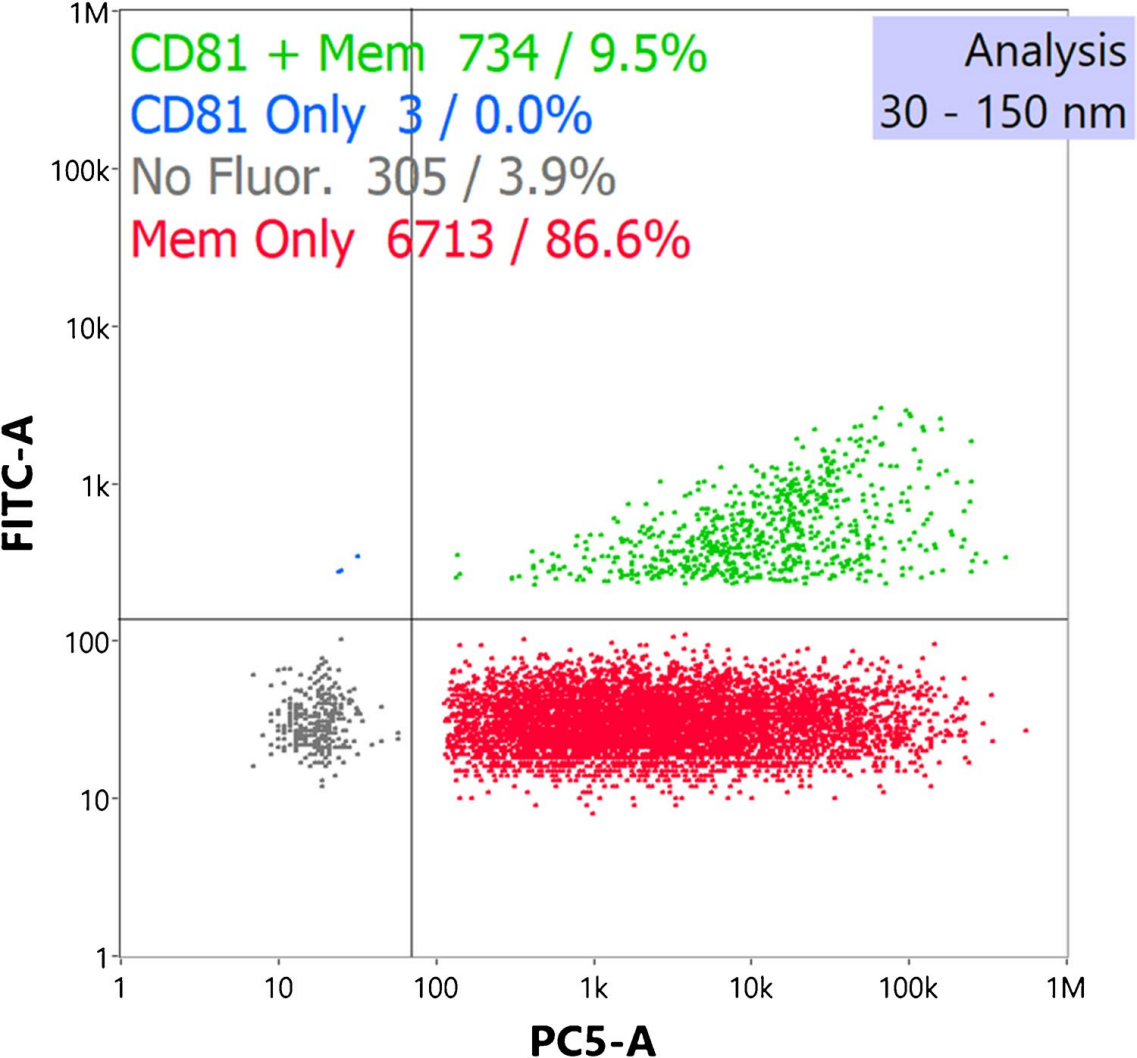
Fluorescent nFCM analysis (*n* = 3) of HIC isolation EVs from HEK supernatant. EVs were labeled with fluorescent anti-CD81 antibodies, as well as the lipophilic membrane-anchored MemGlow dye

Nanoflow cytometry fluorescence detection was employed to detect particle CD81 membrane proteins and phospholipid membranes. In Fig. 4, a typical two-label “four-quadrant plot” is shown, demonstrating the binding of both an anti-CD81 antibody and the Memglow lipophilic membrane dye to the isolated EVs. For this plot, the *x*-axis represents Memglow label events, and the y-axis represents anti-CD81 Ab events.

The summary data in the quad chart reflects the percentages of particles (identified by the side-scattering detector) that yield corresponding fluorescent signals. The minor population (305 events) in the bottom left quadrant corresponds to non-fluorescent events, where unlabeled EVs or background particles are detected via the side-scattering channel. It is important to note that this population has only slightly more events than the control samples, as expected for a population of EVs. The top-left and bottom-right populations represent the Ab-only and Memglow-only labeled populations, respectively, with the final top-right quadrant representing positive fluorescence for both CD81 and Memglow labels. The CD81-only population, comprising just three events, is orders of magnitude less than the Memglow-only or double-positive populations. This disparity is expected as there are only an estimated 1.6 CD81 tetraspanin proteins per EV [62], making it much more likely for EVs to show multiple, positive events per vesicle for the generic membrane dye as opposed to the tetraspanin protein. Future analysis would benefit from the addition of CD9 and CD63 labels for an increased tetraspanin protein signal; however, a ~ 10% positive result is still significant at such a low protein-to-EV ratio. Total particle concentrations from the fluorescently tagged trials were 2.46 × 10^9^ ± 5.5% particles mL^−1^ with ~ 7600 events. This was corrected for the 10 × sample dilution required to maintain events within the manufacturer-recommended levels of 2000–12000 events. Particles were only considered between 30 and 150 nm to remove a minor population of events of high side scatter intensity, likely representative of aggregates. Based on these determinations, it is believed that the HIC C-CP fiber methods for delivering EV isolated from HEK cell supernatants are very well suited for further characterization of size distributions and densities by the three candidate light scattering methods.

Nonfluorescent controls included an elution solvent-only control of 35% ACN in 1X PBS evaporated overnight and an unlabeled EV-only control, evaluated to ensure background fluorescence or EV autofluorescence contributed minimally.

In both cases, the signal from the controls was well below the manufacturer’s recommended event count (< 1000 events), showing only ~ 100 more events than DI H_2_O, with an insignificant background fluorescence signal. Sufficient fluorescent controls are critical for nFCM analysis to analyze label specificity. Fluorescent controls, presented in SI Fig. S4, include (a) fluorescently labeled (anti-CD81 Ab and membrane label) raw HEK supernatant, (b) the protein elution fraction, and (c) neat PBS buffer; all in triplicate. The raw HEK supernatant required significant dilution (1:100 in PBS), with significant populations present in the CD81 + Mem (45.4 ± 3.2%), Mem only (38.7 ± 2.8%), and negative quadrants (42.6 ± 3.3%) (SI Fig. S4a). This is expected with an unprocessed sample, as EVs, lipoproteins, protein aggregates, cell debris, and other matrix components will contribute to the signal. The protein elution control (SI Fig. S4b) would be expected to contain lipoproteins and other proteinaceous species and showed an expected minor positive Memglow signal (125 ± 5 events, only slightly above LOD), which may be attributed to lipoprotein membranes. Importantly, no significant CD81 label signal was detected, suggesting little EV elution in this first step. Finally, the PBS control (SI Fig. S4c) had minimal signal, with an event count similar to H_2_O and a non-fluorescent PBS control (~ 30 events). Neither the CD81 nor the membrane labels had a significant background signal in diluent only.

### LS method comparison of EVs isolated from HEK cell culture supernatant

**Fig. 5:**
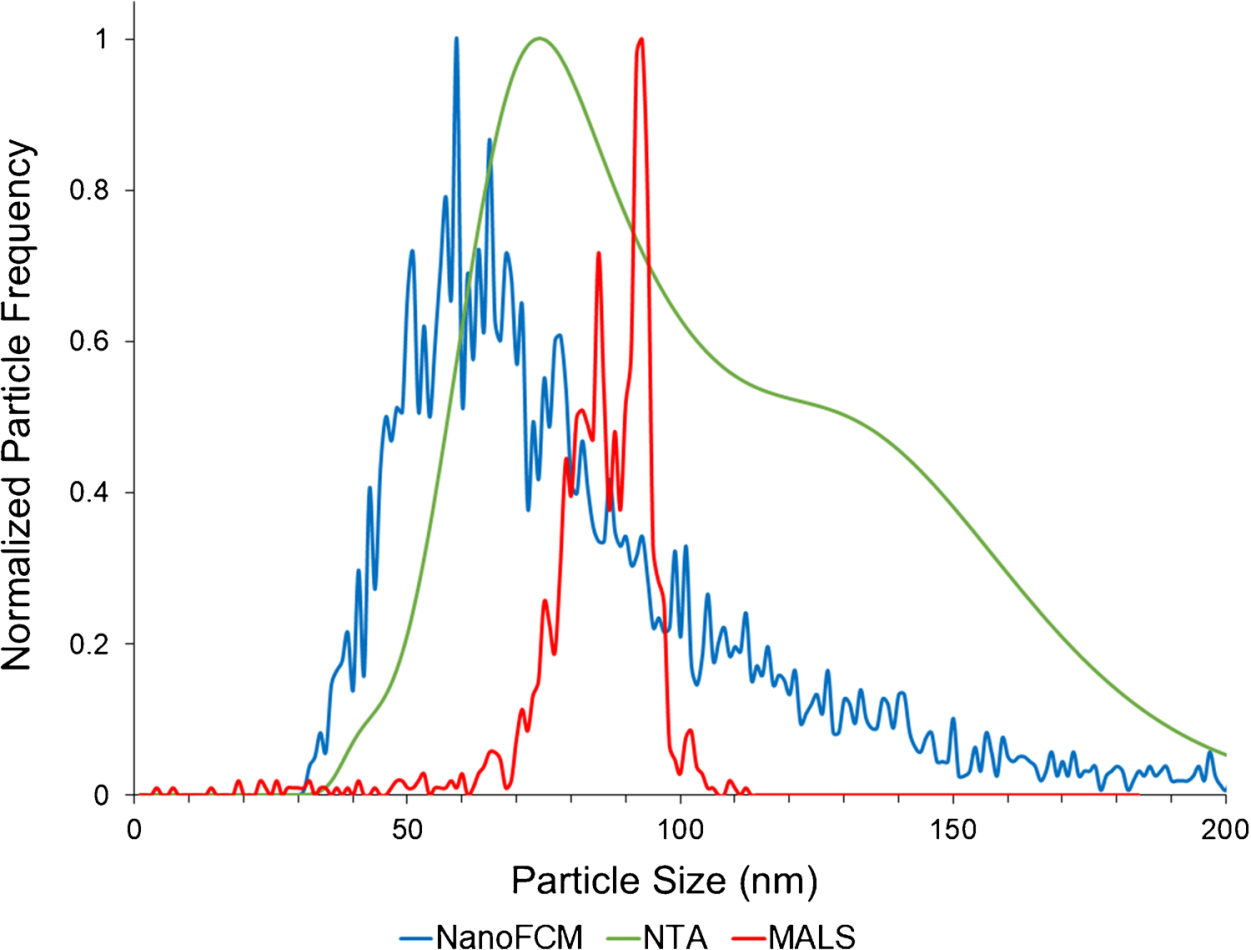
Overlay of average (*n* = 3) particle size distributions (PSDs) derived from the LS instruments for the HIC C-CP fiber isolation of EVs from an HEK293 cell culture supernatant. Note: MALS PSD has been shifted –15 nm based on the results from the single-population standard experiment

As the most practical comparison, EVs isolated from HEK supernatant were analyzed using the three instruments. This specific isolate was obtained from the same primary source as the EV isolate used in the verification trials with UV absorbance, protein removal characteristics, SEM, and nFCM immunoconfirmation. Each instrument analyzed aliquots of this same EV isolate in triplicate (*n* = 3) for the comparison. The average PSDs (*n* = 3) derived from each of the three instruments are presented in Fig. 5 (incorporating the −15 nm shift for the MALS data). The nFCM and NTA both displayed a similar right-skewed (larger size) distribution, from ~ 40 nm to ~ 125 nm in the case of the nFCM and ~ 200 nm for the NTA data. As was the case in the two SiNP test systems, the NTA yielded a broad, smooth distribution, visually suggesting multiple size populations, peaking at ~ 80 and 130 nm, showing a decreasing population above 150 nm. Interestingly, the averaged nFCM distribution, while noisier in structure, also hints towards a heterogeneous size distribution. The presence of multiple size populations is not surprising for the HEK cell source. Very differently, the MALS detector showed a significantly less broad distribution centered between the medians of the other instruments. Again, this particle size averaging is expected from MALS batch mode, highlighting its usefulness in determining average particle sizing in short periods of time (e.g., a chromatographic elution peak) while not providing high definition PSDs.

The quantitative metrics regarding the particle sizing and number density are presented in Table 2 for each of the LS methods. In this dataset, median values are preferable due to the skewing of the nFCM and NTA distributions. As might be suggested visually from Fig. 2, the MALS and NTA measurements generated similar median vesicle size values of 87.2 and 96.8 nm, respectively. Very similar to the 80 nm SiNP standard experiment, the MALS and NTA instruments provided a larger median size value than the nFCM, which provided an appreciably smaller size of 76.0 nm. The consistent bias of the nFCM toward lower median size values is consistent with a previous study from Yan et al., which attributed a ~ 10 nm median negative shift in nFCM sizing to the RI differences between the SiNP calibration standard and EV particles, greatly emphasizing the importance of considering RI differences between standards and samples in nFCM [63]. This may also be due in part to a reduced measurement influence by particle hydration layers as opposed to the NTA and MALS instruments. However, as supported by the performance on the cocktail standard analysis (Fig. 2), the nFCM demonstrated a more accurate particle sizing ability at the lower end of the size range. In addition, these sizing values are consistent with the TEM micrographs of SI Fig. S3, where the observed sizes of 40–150 nm span the range of the nFCM instrument’s comparatively high levels of size resolution.

**Table 2:** Quantitative results among the three light scattering test methods for *n* = 3 determinations of C-CP fiber isolated EVs derived from HEK cell supernatant

Instrument	Avg. diameter (nm)	Median diameter (nm)	Avg. number density (particles mL^−1^)
MALS	86.9 ± 5.4%	87.2	6.40 × 10^9^ ± 4.3%
nFCM	76.7 ± 2.3%	76.0	5.85 × 10^9^ ± 2.9%
NTA	105.1 ± 1.8%	96.8	1.51 × 10^9^ ± 1.7%

The second, and in some cases most relevant, aspect of particle characterization by the LS methods is the determination of particle number densities. To reiterate, each method was calibrated towards density determinations using the SiNP standards. Different from the quantitative results presented in Table 1 for that material, the values presented for the EV isolates are in much closer proximity. In this case, the MALS and nFCM provided very similar number densities (< 10% relative difference) for the HEK supernatant-isolated EVs, while the NTA determination yielded an approximately 4 × lower value. A very interesting and salient feature in the data presented in Table 2, and very consistent with the case for the SiNP standard samples (Table 1), is that each method yielded very good measurement precision in terms of sizing and number density (5% or better). Slight improvements were observed for the MALS and NTA determinations, which may be related to their abilities to better assess the larger particle sizes in the case of the EVs.

To better assess the accuracy of the determined EV concentration values, an EV “standard” was used to estimate the EV concentrations in the original HEK cell culture isolates. This laboratory has demonstrated the efficacy of the use of UV–Vis absorbance as an easily implemented, post-column EV quantification tool [39, 40, 64]. In this case, a fraction of EVs from a similar HEK supernatant sample was isolated, reintroduced to the HPLC (no column injection), and analyzed with UV detection at 216 nm. The average peak area of this measurement was 7.8 ± 1.9% mAU*min (*n* = 3), after a solvent injection background subtraction. The HEK standard was prepared at a slightly higher concentration than expected for the EV isolates, at 1.45 × 10^10^ EVs mL^−1^, and showed a peak area of 11.5 ± 1.8% mAU*min (*n* = 3). Estimating concentration from these peak areas leads to an HEK EV isolate concentration of ~ 9.9 × 10^9^ particles mL^−1^. This value is ~ 2–3 × greater than the values determined by the light scattering methods as presented in Table 2. However, a similar underestimate in number density was also seen in the case of the 82 nm SiNP experiment (Table 1), where the instruments also underestimated by ~ 3–5×. While differences in the isolate supernatant composition could be a reason for the undercounting in the case of EVs, the discrepancy may be more related to the stated concentration in the primary EV standard. Certainly, it is reasonable that this first level of practical comparison was made on a singular extraction method and EV source to establish a baseline. That said, comparison of these methods to EVs from different isolation methods and cell types would be a beneficial future direction to ensure generalization across different EV samples. Overall, each instrument successfully measured the EV concentration within an order of magnitude, with the MALS and nFCM instruments within a 2 × error, performing the best in real sample analysis.

## Conclusions

Three common light scattering detectors were directly compared towards their application in the characterization of EVs, determining their relative capabilities to measure particle sizes spanning the small EV range, while simultaneously measuring the number of particles present. Both single-population and multi-population commercial SiNP standards were used to characterize the baseline performance of the respective methods. Table 3 presents a general overview of the comparison metrics. In general, nFCM was shown to provide better precision and accuracy relative to sizing and quantification, with very clear advantages seen in terms of the size distributions derived from the multicomponent standard. EV samples were prepared from HEK cell supernatant using a PET-Y C-CP fiber column with a HIC separation modality. To support the validity of the LS analysis of real samples, verification of a successful EV separation was determined using multiple techniques, including visualization of EV-fiber binding with SEM, individual vesicle morphology with TEM, immunoconfirmation with anti-CD81 Ab binding and subsequent fluorescent nFCM, and protein removal with Bradford protein assays.

**Table 3:** Summary of the comparison of batch-mode MALS, nFCM, and NTA performance and applicability for EV quantification

Comparator	MALS (batch mode)	nFCM	NTA
Single size standard accuracy and precision	High	High	Moderate
Population resolving power	Broad distributions expected	Capable of subpopulation resolution	Broad distributions expected
Sizing agreement with TEM and SEM	Yes	Yes	Yes
Workflow integration	In-line measurement capable	Off-line	Off-line
Suitability for routine EV analysis	Yes	Yes	Yes
Detection methodology	Bulk-averaged sizing, angle dependence	Single particle scattering intensity	Brownian motion tracking
Data acquisition time	~ 20 min	~ 1–2 h	~ 1–2 h
Primary advantages	Rapid analysis, workflow integration	Fluorescence-equipped, sub-population analysis	Broad sizing range (~ 50–1000 nm)
Primary disadvantages	Bias toward larger particles	Calibration dependent	Bias toward larger particles, greater sample volume needed

The EV isolate purity was ~ 1.8 × 10^9^ EVs µg^−1^ protein, with ~ 9.9 × 10^9^ particles mL^−1^ recovered, with isolation completed within a ~ 10 min separation workflow. The nFCM, MALS, and NTA instruments were able to successfully and reproducibly measure samples and standards with sizes relevant to the EV range, all of which were comparable to TEM and SEM analysis. Particle number density was slightly less consistent instrument-to-instrument, although the MALS and nFCM instruments accurately provided particle densities in cell culture isolate within two times the value of the EV standard, and the NTA instrument was still within an order of magnitude. The MALS instrumentation has the advantage of in-line measurement capabilities, enabling the rapid determination of particle sizing and number density simultaneously with EV chromatographic isolation. This is a specific advantage over the nFCM and NTA, which involve off-line analysis, increasing the workflow time to ~ 1–2 h. Comparing nFCM to NTA, the NTA was overshadowed by the nFCM’s ability to analyze fluorescence simultaneously, both sharing a similar workflow time. These instruments, however, require frequent calibration with sizing standards to ensure instrument accuracy, giving the MALS instrument the advantage of rapid analysis and a greater ease of workflow integration. Overall, the nFCM instrument provided analysis that was superior in terms of accuracy and reproducibility in comparison to the MALS and NTA instruments, with highly accurate PSDs determined for both the single population and multi-population standards. This instrument provided accurate and precise HEK EV sample sizing and number densities, with simultaneous immunofluorescence determinations, within a reasonable workflow burden (< 2 h). Importantly, nFCM also had the ability to measure unlabeled EVs accurately and reproducibly, highlighting its advantages over traditional flow cytometers for EV analysis. Finally, the nFCM, MALS, and NTA instruments all demonstrated a sufficient capability for routine EV analysis, with the MALS instrument providing the most rapid and facile analysis and the nFCM providing the most accurate particle analysis overall, with superior PSDs and accurate particle sizing.

As a final comment, while one would hope that the methods of EV size characterization would be agnostic with regard to the methods of the initial vesicle isolation, it must be admitted that there may be biases. For example, the presence of remnant host cell/matrix proteins, or indeed aggregates thereof, could be problematic. Likewise, the identity/composition of the isolate supernatant could have impacts on the determined characteristics. As such, the quality and modality of the primary EV isolation could certainly impact the performance of any sizing approach and should therefore be evaluated on a case-by-case basis.

## Supplementary Information

Below is the link to the electronic supplementary material.

Supplementary Material 1 (DOCX 644 KB)

## Data Availability

The data that supports the findings of this study are available from the corresponding authors’ upon reasonable request.
