# Supplementary material for: Comparison of light scattering-based detection methods for the sizing and number density characterization of extracellular vesicles (EV) isolated from human embryonic kidney (HEK) cell cultures
Source: Anal Bioanal Chem. 2026 Jan 14;418(11):3177–93. doi: 10.1007/s00216-025-06310-3 (PMC13197382; doi:10.1007/s00216-025-06310-3)
Supplement: Supplementary file 1 — Supplementary Material 1 (DOCX 644 KB) [file 216_2025_6310_MOESM1_ESM.docx]

**Supplementary Information**

**Comparison of light scattering-based detection methods for the sizing and number density characterization of extracellular vesicles (EV) isolated from human embryonic kidney (HEK) cell cultures**

William F. Pons^1, a^, Katelyn M. Joye^2, b^, Terri F. Bruce^2, c^, R. Kenneth Marcus^1, d,^ *

*1 – Department of Chemistry, Biosystems Research Complex, Clemson University, Clemson, SC 29634-0973, USA*

*2 – Clemson Light Imaging Facility, Clemson University, Clemson, SC 29634-0973, USA*

*Corresponding Author: Prof. R. Kenneth Marcus, Department of Chemistry, Biosystems Research Complex, Clemson University, Clemson, SC 29634, USA; marcusr@clemson.edu; ORCID# 0000-0003-4276-5805

Submitted for publication in *Analytical and Bioanalytical Chemistry*


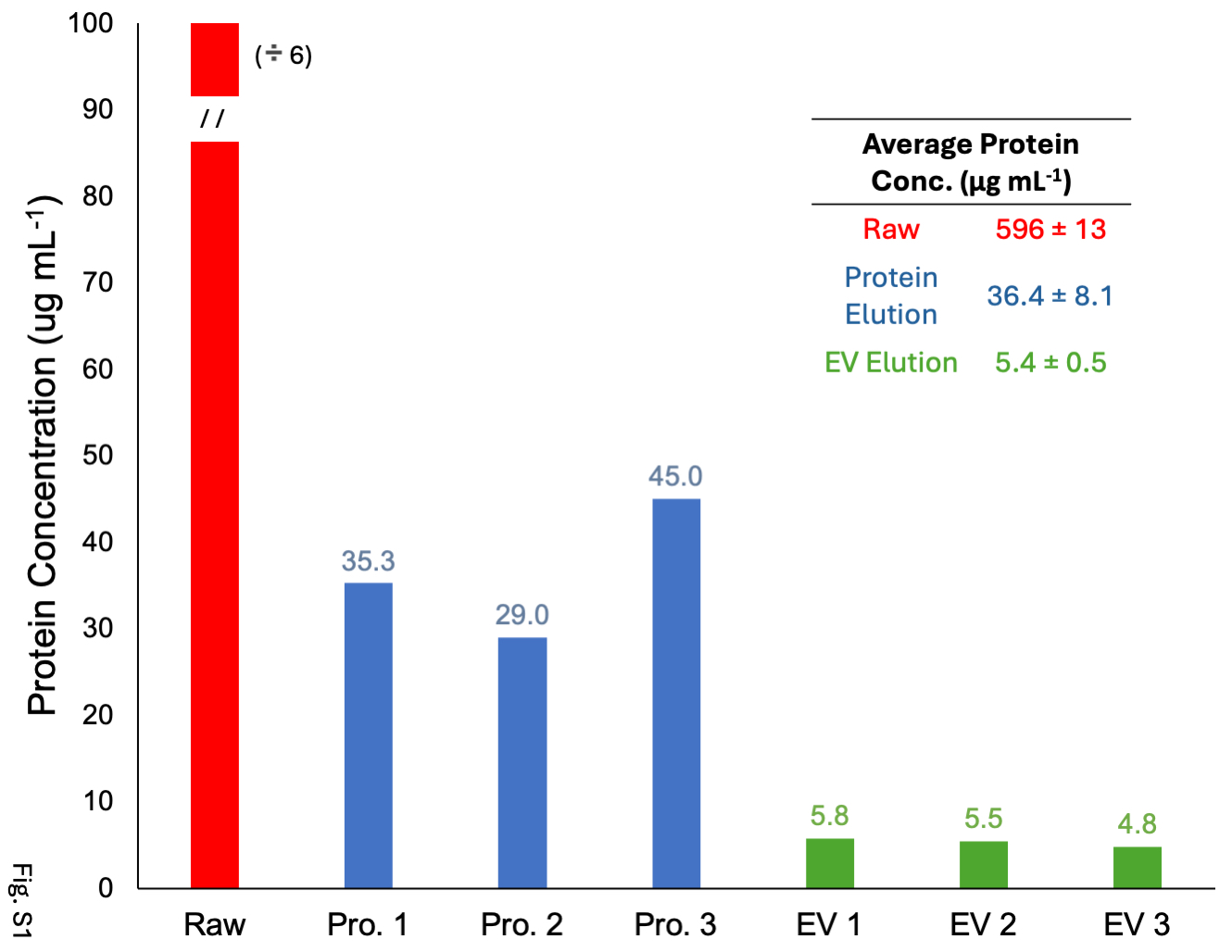


**Figure S1** Results of Bradford assays for n = 3 determinations of the protein/amino acid content at each successive separation step in the isolation of EVs from HEK cell culture supernatant


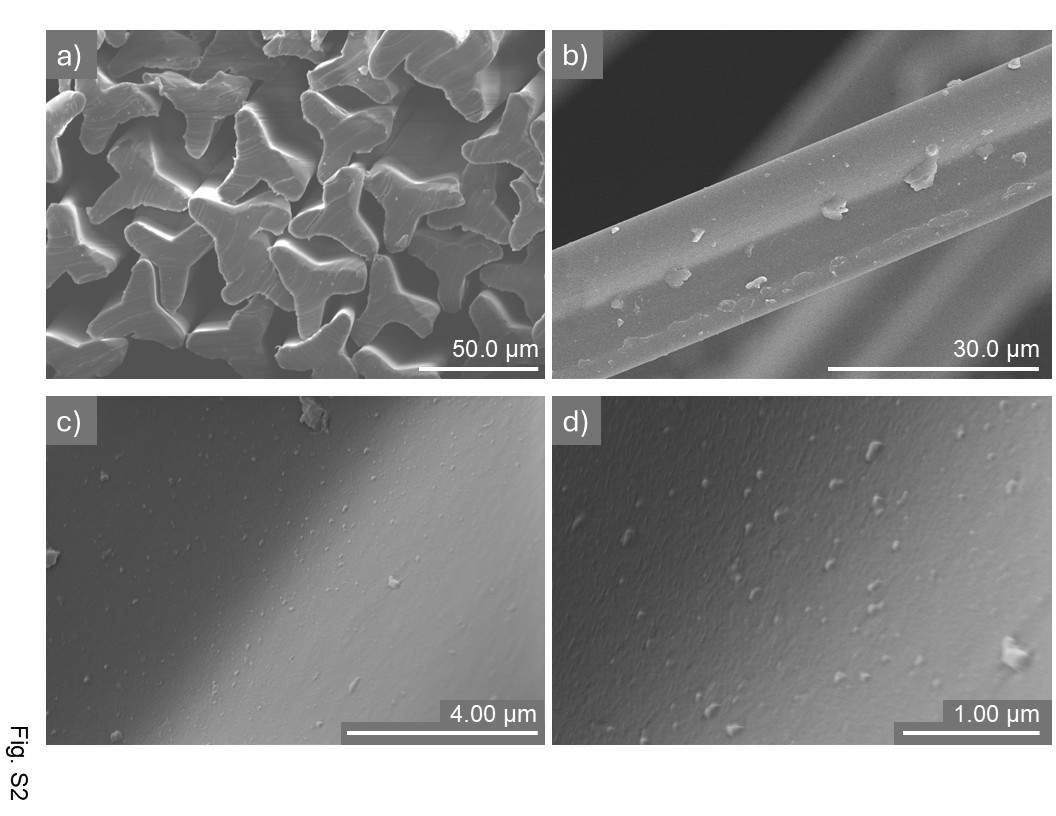


**Figure S2** Scanning electron micrographs of a) PET-Y C-CP fiber column cross-section, and the channels of single PET fibers following EV loading at magnification of b) x1,800, c) x12,000, and d) x35,000


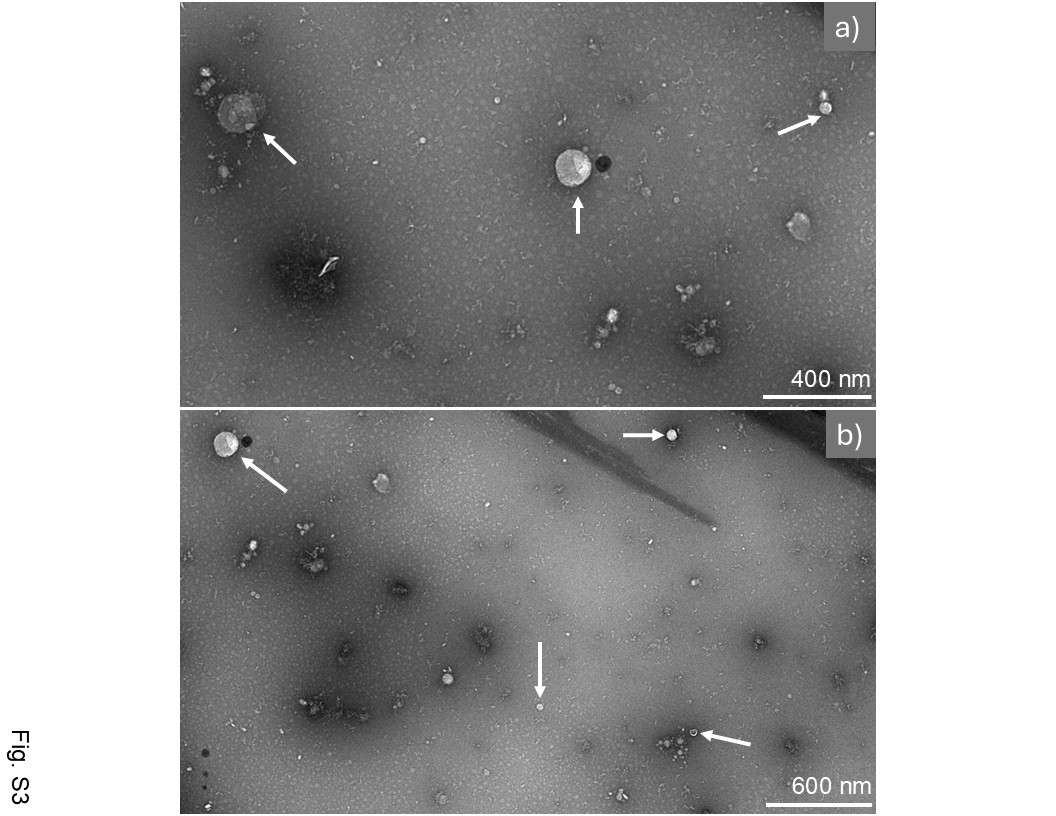


**Figure S3** Transmission electron micrographs of HEK EVs negatively stained with phosphotungstic acid at a magnification of a) 10,000x and b) 6,670x


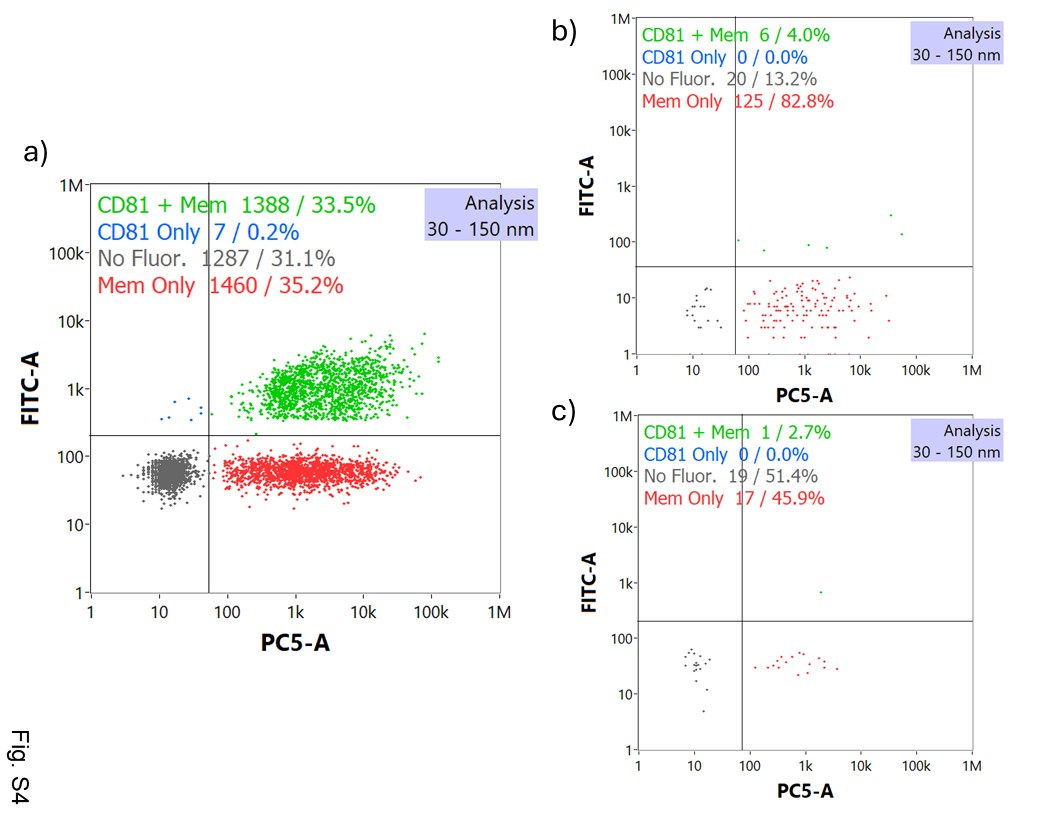


**Figure S4** Fluorescent nFCM control analysis (n = 3) of labeled raw HEK supernatant (a), protein elution (b), and PBS blank (c). All samples were labeled with an identical scheme as Fig. 4, with fluorescent anti-CD81 antibodies and the Memglow lipophilic membrane-anchored dye
